# Effects of an increase in schooling on HIV infection and behavioural risk: a multicountry instrumental variable analysis of 23 sub-Saharan African countries

**DOI:** 10.1136/bmjopen-2025-111925

**Published:** 2026-08-04

**Authors:** Justin Parkhurst, Olivia Klaassen, Pepita Barlow, Ranjeeta Thomas

**Affiliations:** 1 Department of Health Policy, The London School of Economics and Political Science, London, UK

**Keywords:** Africa South of the Sahara, HIV & AIDS, Health policy

## Abstract

**Abstract:**

**Objectives:**

To estimate the causal effect of increased schooling arising from national policy reforms on HIV/AIDS infection and testing, along with associated indicators of risk across a wide range of settings in sub-Saharan Africa.

**Design:**

Multicountry quasi-experimental analysis using two-stage least squares (instrumental variable) analysis with national schooling reforms as the instrument for years of schooling.

**Setting:**

A pooled sample from 23 sub-Saharan African countries with historical peak HIV-prevalence rates ranging from under 1% to over 20%, surveyed from 2003 to 2019.

**Participants:**

A sample of 191 128 cases with linked HIV test results was obtained from nationally representative Demographic and Health Surveys and AIDS Indicator Surveys (and a related survey in Botswana), restricted to adults aged 18–49 years in birth cohorts spanning 1955–1998.

**Primary and secondary outcome measures:**

Primary outcomes were HIV test positivity and ever having been tested for HIV. Secondary outcomes were comprehensive HIV knowledge, age at first intercourse, condom use at last intercourse, number of sexual partners in the preceding 12 months and lifetime number of sexual partners.

**Results:**

Exposure to an education reform increased mean schooling by 0.45 years (95% CI 0.28 to 0.63). Pooled instrumental variable analysis found no significant effect of an additional year of schooling on HIV test positivity (−0.5 percentage points, coefficient −0.005, 95% CI −0.018 to 0.008). One additional year of schooling was associated with a 4.6 percentage point increase in ever having been tested for HIV, significant at the 10% level (coefficient 0.046, 95% CI −0.001 to 0.094). Effects on secondary behavioural outcomes were limited to specific subgroups: women showed a small delay in age at first intercourse while men and urban residents showed increased condom use. No significant effect on comprehensive HIV knowledge was identified in the pooled sample.

**Conclusions:**

Across a wide range of African settings, policies expanding access to schooling appear to increase HIV testing but do not directly reduce HIV infection. Policymakers should look beyond education alone as an HIV prevention strategy, with realistic expectations of schooling expansion as a strategy on its own.

**STRENGTHS AND LIMITATIONS OF THIS STUDY**

- A large pooled sample across 23 countries representing diverse epidemiological contexts greatly expands past one or two country analyses.
- The use of instrumental variable design supports causal analysis.
- Analysis benefits from utilisation of nationally representative survey data with linked HIV test results.
- There was an inability to verify completeness of reform implementation in all country contexts.
- The pooling of primary and secondary reforms may obscure level-specific effects.

## Introduction

 HIV/AIDS continues to be a significant cause of morbidity and mortality internationally, with the 2021 Global Burden of Disease survey estimating it contributes to around 1.4% of global disability-adjusted life-years lost.^1^ The hardest hit region of the world remains sub-Saharan Africa, where over 70% of this disease burden is estimated to lie, and with over one million new infections per year.^1^ While the effectiveness of treatment with highly active antiretroviral therapy (ART) in reducing morbidity, mortality and transmission is clearly established,^2^ the WHO notes that their uptake remains less than optimal, with high HIV infection rates persisting in a number of countries.^3^

The prevention of HIV through policy interventions beyond drug provision thus remains an important global health priority. The theoretical foundations for a causal link between education and health based on the human capital model were first established in the 1970s.^4^ Thus, education is widely seen as a key contributor to improved health and development outcomes in a range of country settings and for a range of health conditions, including HIV/AIDS.^5 6^

There are several potential pathways through which education might affect HIV risk. The first is through increased access to information, which can lead to changes in protective behaviours by affecting perceptions of severity, susceptibility or future opportunity cost.^7 8^ This can reflect general sexual health awareness, as well as HIV/AIDS-specific education (subsequent to the discovery of HIV). Second, schooling may be associated with improved skills, resources and broader capability development to build so-called ‘AIDS resilience’^9^ in any given environment. A third mechanism could be if the school environment constitutes a ‘safe space’ where HIV risk is reduced—as hypothesised in a South African study finding school dropout or lower attendance associated with higher HIV infection and risk.^10^ Finally, a social determinants of health perspective^11^ recognises education leads to individuals becoming embedded in different settings and contexts (eg, through employment, place of residence, social networks)—with differing associated patterns of risk.^12^

However, the direct causal impact of education on HIV/AIDS infection can be difficult to assess, particularly through observational analyses that have led to seemingly contradictory results in the past. In 2002, a systematic review found education to be associated with higher HIV rates in African countries, although it was found to be protective in Thailand.^13^ A 2009 multicountry cross-sectional analysis found education to be associated with increased condom use and use of testing, but also higher reported rates of marital infidelity and lower rates of abstinence.^14^ In 2017, a cross-sectional analysis of four African countries argued that education may be associated with higher risk of infection in early years but become protective as epidemics evolve.^15^ More recently, another cross-sectional analysis of 32 sub-Saharan African countries found secondary school completion associated with lower rates of positive HIV tests in females, while primary completion was associated with higher rates for males and females.^16^ Observational studies, however, face a number of challenges of potential confounding with other determinants of HIV risk that may be unaccounted for—for example, parental education, attitudes towards risk, mobility and migration.

These challenges highlight the need for causal analyses of this question. To date, however, there have been limited studies of this nature. Randomised trials have mostly focused on interventions in specific subpopulations or local areas which aim to increase schooling through cash transfers or lotteries, with some HIV-related outcomes assessed. For instance, Duflo *et al* undertook a randomised trial in Western Kenya that reduced the cost of education, finding it led to lower rates of teenage childbearing.^17^ Baird *et al* alternatively undertook a multiarm trial in one region of Malawi, and found lower rates of HIV and HSV-2 in intervention groups receiving a cash transfer, but no significant difference between those for whom the transfer was conditional on school attendance versus unconditional.^18^ Finally, Gorgens *et al* found significantly lower HIV prevalence for adolescent girls and young women (age 15–22) after giving a financial incentive conditional on education participation in Eswatini.^19^

We are aware of only three other causal analyses of this question with national population-wide scope—all of which used a national education reform policy as an instrument to consider variation in levels of schooling to study HIV outcomes. The use of instrumental variable (IV) methods, however, has been widely used in this way to analyse causal relationships between schooling and a range of other health and social outcomes in low and middle income settings, including: child mortality in Zimbabwe,^20^ Uganda and Malawi^21^; child health in Turkey^22^; overweight/obesity^23^ or female genital cutting^24^ in Nigeria; and early child marriage in China.^25^

The fundamental logic of these analyses is that a national reform that shifts amounts of schooling for some cohorts (those born late enough to benefit) creates exogenous quasi-experimental variation in schooling. Comparing otherwise-similar ‘treated’ and ‘control’ cohorts (defined by birth year) isolates the effects of schooling on the health outcome, free from the confounding issues concerning observational studies—provided there are no other relevant differences between populations born in the treated and control cohorts.

Behrman^25^ considered the impact of universal primary education reforms on HIV rates in women in Uganda and Malawi, while De Neve *et al*^26^ looked at the HIV impact of a policy mandating a 10th year of education in Botswana. The first of these found a 1-year increase in girls' schooling led to a decrease in women testing HIV positive by 6 or 3 percentage points (pp) in Malawi or Uganda, respectively; while De Neve *et al* found that each additional year of schooling led to a reduction of HIV by 8.1 pp. These have been widely cited due to the very large impacts reported, but the Botswana findings could not be reproduced by a later expanded analysis by Lindskog and Durevall.^27^ These authors only identified a significant reduction in HIV prevalence for young women (18–24), with no significant impact at all on men or older women.^27^

Individual studies not only can face replicability issues as shown, they also may present external validity challenges across contexts. Botswana has persistently reported one of the world’s highest prevalence rates, peaking at over 25% (from 1999 to 2001); while Uganda and Malawi also have relatively high prevalence rates peaking at 9.4% (1991–1992) and 15.1% (1998), respectively (data from aidsinfo.unaids.org). These previous studies also considered different policies: a secondary education reform in Botswana and primary education reforms in Uganda and Malawi analysing women only.

As such, strengthening the evidence base of causal studies is crucial, particularly given how important they can be to inform policy advocacy and decision-making. After the first Botswana analysis was published, Remme *et al* argued that expanding schooling could be ‘as good an HIV investment as male circumcision’.^28^ Similarly, recently, UNESCO quoted a senior official as stating that “evidence show[s] that each additional year of secondary schooling leads to a reduction in the cumulative risk of HIV infection”^29^—potentially based on the studies showing large positive results. Furthermore, the language of education as a ‘social vaccine’ for HIV is commonly presented in global health scholarship and advocacy, at times appearing to take for granted that an extra year of schooling will (on its own) reduce HIV incidence.^15 30^

To provide a broader evidence base for this issue, our objective was to identify and analyse data from as many countries as possible which have had universal education reforms along with comparable HIV testing and behavioural survey data to perform causal analysis across a much wider range of settings. Our study ultimately analyses pooled data from 23 African countries representing low, medium and high prevalence environments where national peak prevalence rates ranged from under 1% to over 20%. We look both at the effects of increased schooling on HIV status (test positivity) and on having ever been tested for HIV (a critical factor to increase treatment rates), but also on a set of potential causal factors related to knowledge and behaviour. We also include consideration of male/female sex and rural/urban residence in our analysis.

## Methods

This was a multicountry quasi-experimental (IV) analysis of pooled, nationally representative cross-sectional survey data from 23 sub-Saharan African countries. Data were obtained from Demographic and Health Surveys (DHS) or related AIDS Indicator Surveys (AIS) conducted between 2003 and 2019, as well as a similar survey in Botswana. National education reforms were used as an instrument for years of schooling to estimate causal effects on HIV outcomes for adults aged 18–49.

### Country identification

We began with a literature review and scoping search to identify countries which had undertaken educational reforms at a national level which could serve as an instrument for the analysis. We limited inclusion to: universal primary (or basic) education, universal secondary education, compulsory schooling laws, increased years of schooling and elimination of tuition fees. We cross-referenced literature review results against World Bank reports on universal education to check type of reform and the date of adoption. Where unclear, we undertook individual searches for country-specific information. We then compared our findings with the Stanford University World Education Reform Database^31^ which provides a table list of countries and education reforms (although often lacking significant detail). We excluded countries where the only adequate education reform occurred before 1970, predating the HIV pandemic by too many years or where implementation was gradual or unclear. From this shortlist of countries, we then identified those where HIV testing data were available from one or more nationally representative DHS or related AIS surveys (and a similar survey in Botswana). Greater explanation of our strategy can also be found in online supplemental appendix.

We excluded countries where the youngest cohort impacted by the education reform would be under 18 when surveys were conducted. Ultimately, we identified 25 potential countries for inclusion, 23 from sub-Saharan Africa plus two in Asia (India and Vietnam). Given that neither Asian country ever reported generalised epidemics of prevalence over 1%, we decided to exclude these two countries from the final sample. The survey datasets themselves have been publicly available until recently, although they require registration and additional permission requests for HIV data. Before 2025, data could be obtained from the MeasureDHS programme: https://dhsprogram.com/; or for Botswana, the data used came from Statistics Botswana: https://microdata.statsbots.org.bw. At the time of writing, the DHS website indicates that the DHS programme was ‘on pause’ and no additional datasets could be requested, although existing ones could still be downloaded if previously approved.

### Patient and public involvement

This study uses publicly available secondary data, pooled from a large number of countries. Countries were not chosen in advance, but rather included based on availability of policy reforms and linked data. As such, there was no direct involvement of the public in relation to the analysis undertaken.

### Variables of interest and cohort construction

Our primary outcomes are the respondent’s HIV status at the time of the survey and HIV testing, based on reported ‘ever been tested’ for HIV. Secondary outcomes relate to potential mechanisms: these include a measure of ‘comprehensive HIV knowledge’ and variables which indicate self-reported HIV-related behaviours. Comprehensive knowledge is defined by DHS as participants responding ‘yes’ to: condoms prevent AIDS transmission, ‘yes’ to healthy looking persons can have AIDS, and ‘no’ to the two most common local myths (country specific). HIV behaviour variables included: age at first intercourse, condom use during last intercourse, two or more sexual partners in the last year and lifetime sexual partners.

We defined our ‘exposed’ to policy reform group as those of an age expected to benefit from the country-specific reform. The oldest of this cohort were those who would be in the final year of schooling affected by the policy (either primary or secondary). These individuals, in addition to those born in the following 5 years constituted the ‘exposed’ group. Following previous IV analyses,^20 21 23^ we imposed a gap between the earliest birth year in the exposed group (oldest exposed individual) and the latest birth year of the ‘control’ cohort (youngest control individual) to reduce partial exposure due to the possibility of late initiation of schooling and/or grade repetition (we use a 3-year gap and reduce this in sensitivity analysis). Thus, our control group starts with birth cohorts born 4 years before the oldest exposed birth cohort and includes those born in the preceding 5 years. To provide an illustrative example: Cameroon eliminated primary school fees in 2000 and students are expected to complete primary school at the age of 12. Therefore, the exposed cohort for Cameroon was defined as a 6-year group who would reach their 12th birthday, or younger, in 2000: born between 1988 and 1993. The control cohort was constructed using ages assumed too old to have benefited from this reform. In Cameroon, applying a 3-year exclusion to avoid partial exposure, the control group consisted of individuals born between 1979 and 1984. In addition, given our focus on HIV, we restrict our sample to individuals within these cohorts who are between 18 and 49 years of age when surveyed. Table 1 provides the specific details of reforms and cohorts constructed for all countries included.

**Table 1:** Country education reform details, sources and cohorts implied

Country	Reform type	Policy implemented	References	Reform-affected school exit age	Exposed cohort birth years	Control cohort birth years
Angola	Compulsory free primary education	2001	^36^	11	1990–1995	1981–1986
Botswana	Additional compulsory year of secondary education	1996	^26^	15	1981–1986	1972–1977
Burundi	Primary school fee removal	2005	^36–38^	12	1993–1998	1984–1989
Cameroon	Primary school fee removal	2000	^37^	12	1988–1993	1979–1984
Ethiopia	School fees to year 10 eliminated	1995	^36 37^	16	1979–1984	1970–1975
Gambia	Compulsory free primary education	1998	^39 40^	12	1986–1991	1977–1982
Ghana	Fee abolition for ‘basic school’	1996	^36 41 42^	14	1982–1987	1973–1978
Kenya	Free primary education for years 1–4	1974	^36 43–46^	10	1964–1969	1955–1960
Lesotho	Primary school fees eliminated	2000	^37 47^	12	1988–1993	1979–1984
Liberia	Primary school fee abolition	2006	^36^	12	1993–1998	1984–1989
Malawi	Elimination of primary school fees	1994	^21 36^	13	1981–1986	1972–1977
Mali	Compulsory and free schooling	1993	^36 40 48 49^	14	1979–1984	1970–1975
Mozambique	End of civil conflict and free primary education policy	1995	^36 50^	12	1983–1988	1974–1979
Niger	Universal basic (primary) education	1998	^36^	12	1986–1991	1977–1982
Republic of Congo (Brazzaville)	Compulsory schooling up to age 16	1992	^40 51^	15	1977–1982	1968–1973
Rwanda	Tuition fees eliminated	2003	^37 52 53^	12	1991–1996	1982–1987
Senegal	Compulsory schooling law	2004	^40 54^	16	1988–1993	1979–1984
Sierra Leone	Compulsory schooling laws–9 years basic education to be free	2004	^40 55 56^	14	1990–1995	1981–1986
South Africa	Compulsory schooling law	1996	^40^ and RSA government website	15	1981–1986	1972–1977
Tanzania	Fee removal for primary education	2002	^57^	13	1989–1994	1980–1985
Uganda	Free primary education	1997	^21 53 58 59^	12	1985–1990	1976–1981
Zambia	Tuition fees eliminated	2002	^37 59 60^	13	1989–1994	1980–1985
Zimbabwe	Free compulsory primary education, automatic progression from primary to secondary and dramatic expansion of secondary education.	1980	^20 61 62^	16	1964–1969	1955–1960

### Statistical analysis

The primary estimand of this study is the average causal effect of an additional year of schooling on HIV positive status and testing across sub-Saharan Africa. To this end, we adopt a pooled cross-country ordinary least squares (OLS) and IV design that exploits variation in the timing of national schooling reforms across 23 countries as a source of exogenous variation in schooling. This approach follows an established tradition in the education and health literature of using cross-country reform variation to identify generalised causal effects. All models are estimated with robust standard errors clustered by country and year of birth. Models were estimated without survey weights, as our estimand is the local average treatment effect among reform-affected cohorts rather than a population-representative mean (following the approach of De Neve *et al*,^26^ and Behrman).^25^ The first OLS model provides a descriptive association between years of schooling and HIV status. We then also estimate an intention-to-treat (ITT) parameter using OLS. This captures the impact of being exposed to education reforms on HIV/AIDS outcomes. While this analysis addresses the potential endogeneity present in the first OLS model, it does not capture the impact of an additional year of schooling on the outcomes.

Our OLS models linking outcomes to an individual’s years of schooling are specified as follows:


$$\begin{array}{l} H_{i}=\beta_{0}+\beta_{1}S_{i}+i.{male}_{i}\;\#\#\;(\beta_{2}age_{i}+\beta_{3}age_{i}^{2}+\mu_{country}\\ +\delta_{country\times cohort}+\nu_{cohort}+\lambda_{survey}+\epsilon_{i}) \end{array}$$

Where $H_{i}$ is a binary variable representing one of our outcomes for individual *i*. $S_{i}$ denotes an individual’s years of schooling. age and $age^{2}$ capture potential non-linear age effects. $male_{i}$ is a binary indicator of an individual’s sex. $\mu_{country}$ and $\lambda_{survey}$ are country and survey year fixed effects accounting for country differences and survey year differences, respectively. $\nu_{cohort}$ captures a global secular birth cohort trend. We also include a country-specific linear trend in birth cohorts. These trends are intended to capture country-specific improvements in health that have occurred independent of the education reforms, or even macroeconomic factors which may positively affect education reforms and household resources which in turn may influence health outcomes. For our ITT model, we replace *S_i_* with a binary indicator of treatment.

**Figure 1:**
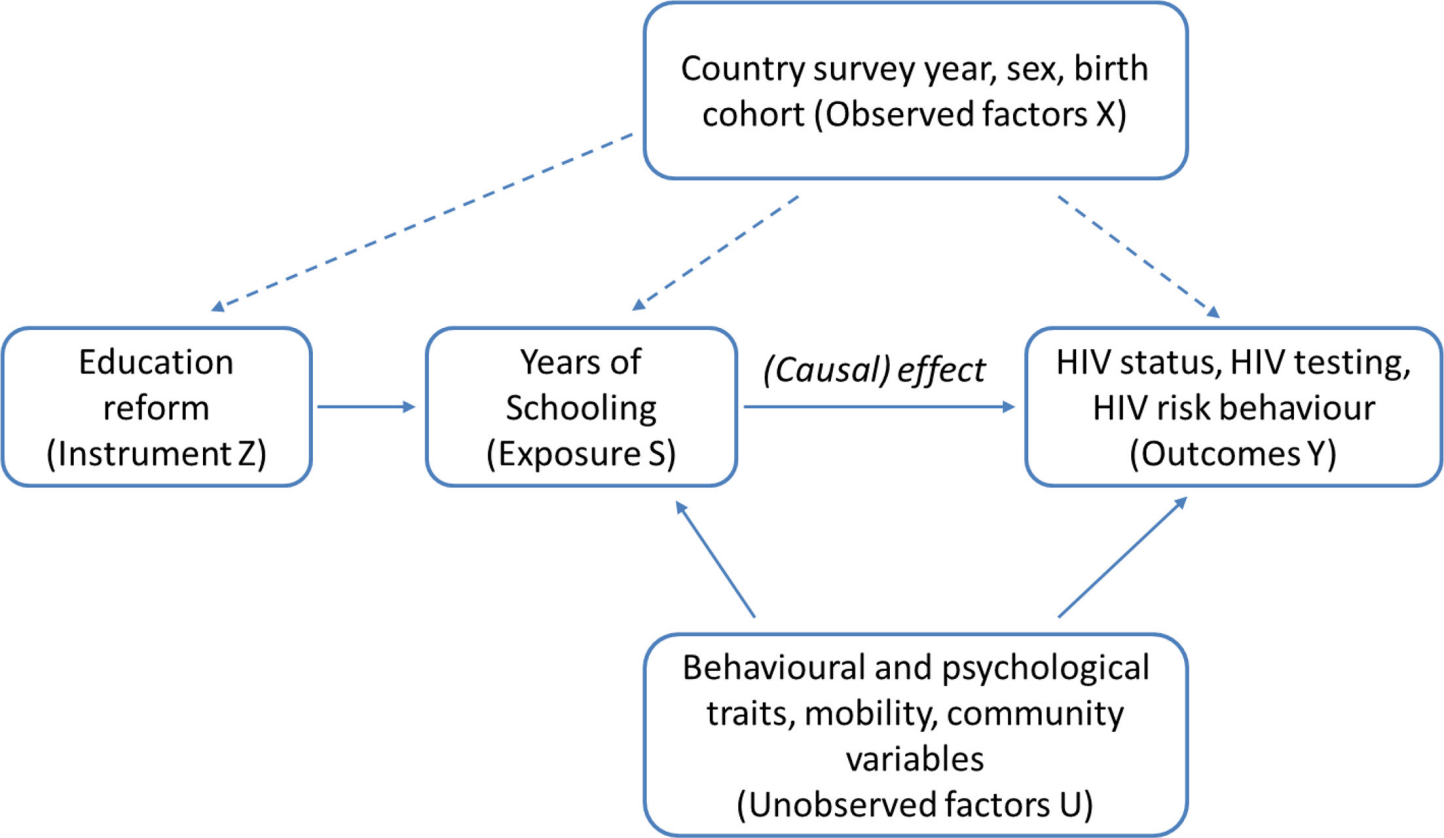
Causal Diagram – main analysis. Source: adapted from De Neve et al. (2015) – https://www.thelancet.com/journals/langlo/article/PIIS2214-109X%281500087-X/fulltext Reproduced in accordance with the Creative Commons (CC-BY) Open Access licence https://creativecommons.org/licenses/by/3.0/.

Following previous authors’ analyses,^23 25 26^ we use IV models in our main analysis to estimate the local average treatment effect (LATE) of one additional year of schooling.^23 26^ The analysis uses the policy reform as an instrument for years of schooling, allowing us to deal with the issue of endogeneity of schooling, and thus estimate the causal impact of an additional year of schooling on HIV outcomes. The identifying assumption is that, conditional on adjustment variables, the policy reform is uncorrelated with HIV outcomes, except through its effect in increasing years of schooling; and independent of any unobserved determinants of HIV outcomes. The approach also assumes monotonicity, that is, the reforms did not lead some recipients to obtain fewer years of schooling. A graphical illustration of the assumptions of causality in the IV analysis is presented below (figure 1) in a reproduction (with adapted text) of the directed acyclic graph produced by De Neve *et al* demonstrating assumptions of the analysis. The instrument of the education reform is assumed valid if it affects HIV risk only through changes in years of schooling and is assumed to be uncorrelated with unobserved factors (U).

For our IV models we estimate the following first and second stage equations using two-stage least squares (2SLS):


$$\begin{array}{l} S_{i}=\beta_{0}+\beta_{1}{\text{exposed}}_{i}+i.{male}_{i}\#\#(\beta_{2}age_{i}+\beta_{3}age_{i}^{2}+\rho_{country}\\ +\kappa_{country*cohort}+\tau_{cohort}+\gamma_{survey}+\omega_{i}) \end{array}$$

$$\begin{array}{l} H_{i}=\beta_{0}+\beta_{1}{\hat{S}}_{i}+i.{male}_{i}\;\#\#\;(\beta_{2}age_{i}+\beta_{3}age_{i}^{2}+\mu_{country}\\ +\delta_{country\times cohort}+\nu_{cohort}+\lambda_{survey}+\epsilon_{i}) \end{array}$$

To assess impact by subgroups of sex and urban/rural location, we estimated IV models interacting the treatment indicator with each of these variables. We report the LATE for each subgroup (ie, men/women; urban/rural).

### Robustness checks

To test the robustness of the IV results, first, we repeated the analysis on a sample limited to surveys conducted from 2010 onwards. This ensures inclusion of only mature HIV epidemics and population-wide access to ART. Testing refusal rates in the surveys can also fall over time in most countries, so later surveys may capture lower refusal rates (see online supplemental appendix table S1). This left 29 rounds of surveys conducted within 20 countries for this check. Second, we re-estimated models with a smaller 2-year gap window to reduce temporal differences between the exposed and control cohorts (but risking more partial exposure). We also re-ran the primary analysis on a subsample of countries with peak prevalence rates over 10% (listed in online supplemental table S5) to restrict analysis exclusively to the highest prevalence settings.

Our primary analysis estimates linear probability models via two-stage least squares in line with the previous causal analyses of this question by De Neve *et al*^26^ and Behrman.^25^ While the linear probability model is standard in this literature and produces coefficients interpretable as pp changes in probability, we additionally utilised an IV probit model for all binary outcomes as a robustness check. The probit models were estimated using Newey’s two-step estimator^32^ rather than maximum likelihood, as the latter was computationally infeasible given the sample size and model complexity.

We also include a forest plot of country-specific IV estimates as supplementary figures for our two key outcomes—HIV positive result and HIV testing (online supplemental appendix figures S1, S2). These country-specific estimates are not the primary estimands of the paper—the small sample sizes available within individual countries after cohort restriction mean that we expect many estimates to be imprecise, with wide CIs that preclude meaningful country-level inference. Furthermore, it is possible that specific schooling reforms may not always generate sufficient variation in schooling within these country-specific samples to support reliable IV estimation. Rather, these forest plots are presented as supplementary evidence to assess whether the pooled result reflects a broadly consistent pattern across countries or is driven by a subset of countries. Finally, we present adjusted p values correcting for multiple hypothesis testing across two families of hypotheses: (1) HIV positive status and HIV testing and (2) across our five behavioural outcomes.^33^

## Results

Table 2 provides summary statistics of our included pooled population:

**Table 2:** Summary statistics

Variables	Total		Exposed		Control	
	Observations	Mean (SD) or proportion	Observations	Mean (SD) or proportion	Observations	Mean (SD) or proportion
Stage 1. Dependent						
Total and % ‘exposed’ to an education reform	197 657	49.37%				
Stage 2. Dependent						
Total number of years of education	195 185	5.95 (4.55)	96 267	6.57 (4.24)	98 918	5.13 (4.33)
Stage 2. Independent						
HIV-positive test result	191 128	7.00%	94 490	6.00%	96 638	8.00%
Ever been tested for HIV	194 434	51.04%	96 194	53.00%	98 240	49.00%
Comprehensive HIV knowledge	148 039	48.22%	74 381	48.00%	73 658	48.00%
Age at first intercourse (in years)	162 731	17.44 (3.35)	76 155	17.06 (2.97)	86 576	17.78 (3.62)
Condom used at last intercourse	147 024	16.70%	66 602	20.00%	80 422	14.00%
Had 2 or more sexual partners in 12 months	193 688	12.47%	95 118	12.00%	98 570	13.00%
Number of sexual partners in lifetime	147 113	3.69 (7.84)	70 365	3.51 (7.51)	76 748	3.86 (8.13)
Baseline characteristics						
Sex (% male)	196 444	42.09%	96 889	42.00%	99 555	42.00%
Age (in years)	197 657	26.93 (6.35)	97 580	24.01 (5.55)	100 077	29.69 (5.86)
Urban/rural status (% urban)	191 539	36.64%	94 124	39.00%	97 415	35.00%

Binary variables are presented as percentages. SDs in parentheses. Unweighted data.

The full sample included 197 657 individuals across 23 countries. 49.37% were in the exposed cohort based on cohort construction. Mean years of schooling was 5.95 years in the full sample; 6.57 in the exposed group and 5.13 in the control group. 191 128 (96.69%) individuals had linked HIV test results, of whom 7.00% were HIV-positive. OLS regression results show a very small increase in HIV positivity of 0.1 pp (coefficient 0.001, 95% CI 0.000 to 0.001) associated with schooling, while the ITT analysis shows no significant effect on HIV positivity (−0.2 pp, coefficient −0.002, 95% CI −0.009 to 0.004) from exposure to reform (table 3).

**Table 3:** Results on HIV status and HIV testing

	Exposure	Outcome	Effect estimate	(Coefficient, 95% CI)
All participants (n=191 128 for model 3, second stage)				
Model 1: OLS	Years of schooling	HIV positive	0.1 percentage points	(0.001, 0.000 to 0.001)
Model 2: intention to treat	Benefit from reform	HIV positive	−0.2 percentage points	(−0.002, −0.009 to 0.004)
Model 3 (2SLS): first stage	Benefit from reform	Years of schooling	0.454 years	(0.454, 0.282 to 0.625)
Model 3 (2SLS): LATE	Additional years of schooling from reform	HIV positive	−0.5 percentage points	(−0.005, −0.018 to 0.008)
Model 3 (2SLS): LATE	Additional years of schooling from reform	Ever been tested for HIV	4.6 percentage points	(0.046, −0.001 to 0.094)

LATE, local average treatment effect; OLS, ordinary least squares; SLS, stage least square.

**Figure 2:**
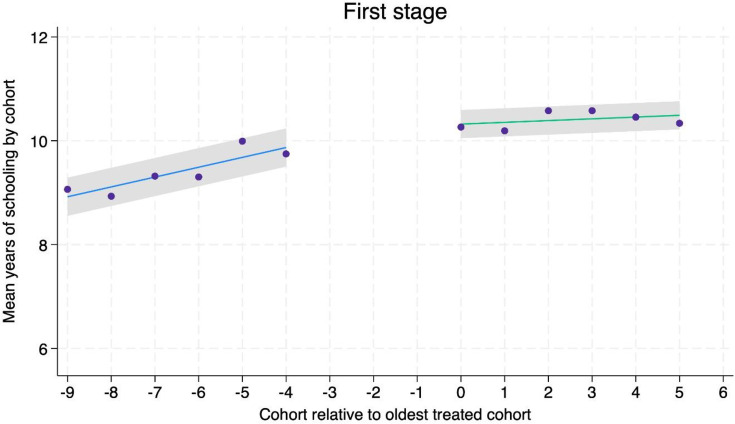
Years of schooling achieved. Notes: Figure 2 shows mean years of schooling in relation to birth year relative to the oldest-treated birth year (country specific) set to 0 on the X axis – thus negative numbers represent earlier birth years (and thus the control cohort) and positive numbers represent later birth years (exposed to the reform). The shaded area shows the 95% Confidence Interval. The figure illustrates the three-year exclusion gap utilised in the primary analysis.

**Figure 3:**
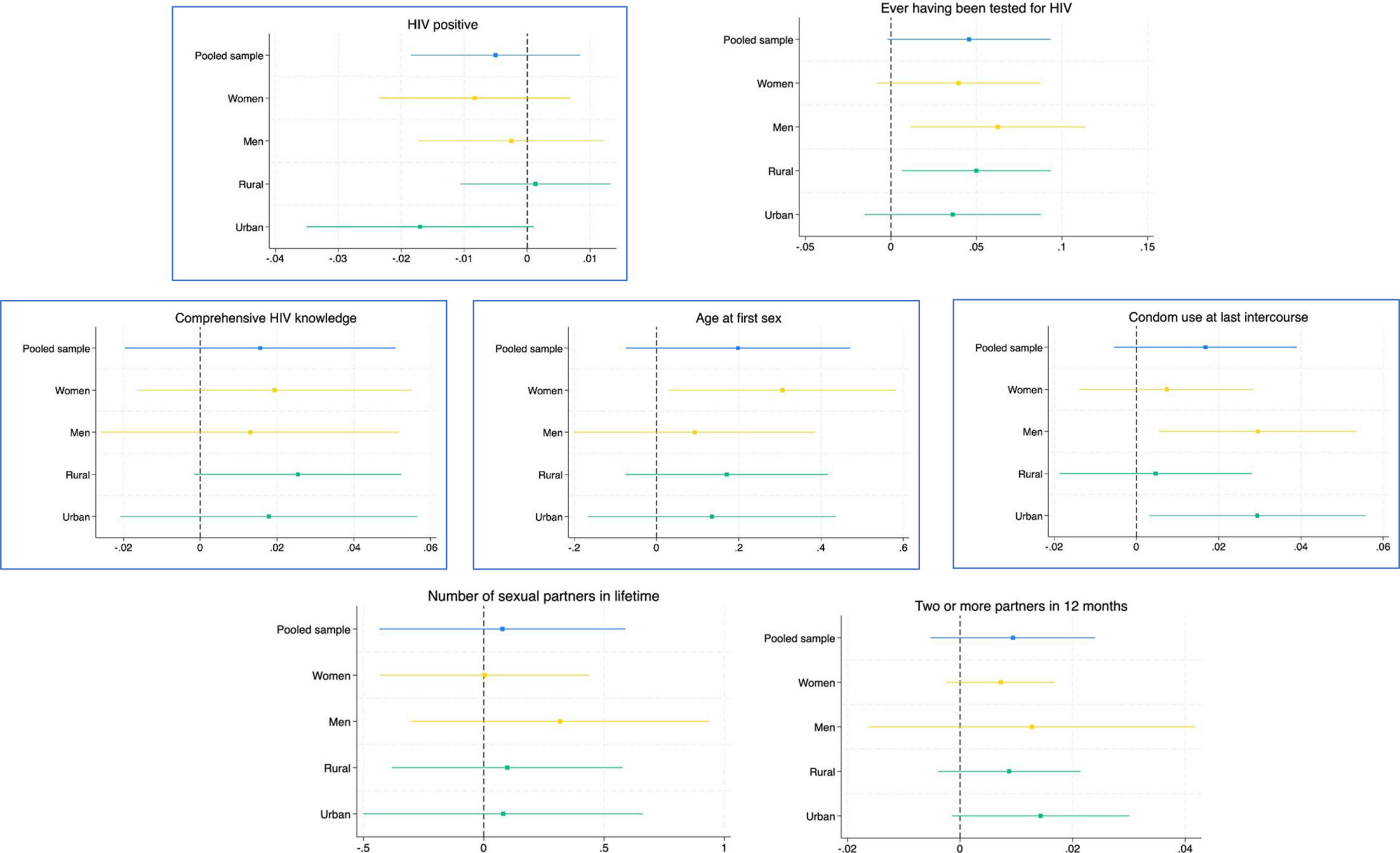
Primary and Secondary Results – 2 stage least squares results with 95% confidence intervals*. *Note - Data populating graphs provided in the online supplemental appendix.

The first stage of the 2SLS model shows being exposed to an education reform resulted in an average increase of 0.454 years of schooling (95% CI 0.282 to 0.625) figure 2 and table 3). Thus, exposure to reform significantly predicted years of schooling, with first-stage F-statistics ranging from 18.9 to 27.1 across models (table 4), well above the conventional threshold of 10 used to indicate a strong instrument. The second stage (LATE) results from the pooled model show no statistically significant effect of an additional year of schooling on HIV test positivity (−0.5 pps, coefficient −0.005, 95% CI −0.018 to 0.008). However, one additional year of schooling increased HIV testing by 4.6 pp, significant at the 10% level (coefficient 0.046, 95% CI −0.001 to 0.094) (see figure 3 and table 4). In the pooled models, we do not find significant effects of more schooling on behavioural outcomes.

**Table 4:** Local average treatment effect of additional year of schooling in the pooled sample—second stage estimates

Model number	(1)	(2)	(3)	(4)	(5)	(6)	(7)
Outcome	HIV positive	Ever been tested for HIV	Comprehensive HIV knowledge	Age at first sex	Used condom at last intercourse	2 or more sexual partners in 12 months	Number of sexual partners in lifetime
Units (pp=percentage point)	pp	pp	pp	years	pp	pp	number
Years of schooling	−0.5	4.6*	1.6	0.198	1.7	0.9	0.078
SE	(0.7)	(2.4)	(1.8)	(0.139)	(1.1)	(1.0)	(0.375)
Age	✓	✓	✓	✓	✓	✓	✓
Age squared	✓	✓	✓	✓	✓	✓	✓
Sex	✓	✓	✓	✓	✓	✓	✓
Birthyear trend	✓	✓	✓	✓	✓	✓	✓
Country fixed effects	✓	✓	✓	✓	✓	✓	✓
Survey year fixed effects	✓	✓	✓	✓	✓	✓	✓
Sex×all covariates	✓	✓	✓	✓	✓	✓	✓
N	190 116	193 287	146 925	161 745	146 508	192 730	147 039
F-statistic	27.038	27.112	22.452	18.944	21.391	26.058	21.548

Robust SEs are clustered at the country-cohort level in parentheses.

*p<0.10.

When considering subgroups, we find no impact on HIV status by sex or location (figure 3 and online supplemental appendix tables S2, S3). We find a significant increase in HIV testing among rural residents of 5 pp (coefficient 0.05; 95% CI 0.006 to 0.093). Considering subgroups among the behavioural outcomes, we see a small increase in age at first sex among women of 0.316 years (95% CI 0.036 to 0.597); and condom use among men of 5.5 pp (coefficient 0.055, 95% CI 0.005 to 0.105) and urban residents of 3.0 pp (coefficient 0.030, 95% CI 0.004 to 0.057).

### Robustness checks

The primary results from the pooled models are robust to restricting the analysis to post 2010 surveys (table 4). Narrowing the gap between cohorts to 2 years results in loss of significance of primary results on testing, potentially due to partially exposed population now included as controls. The only significant secondary outcome in this check is a slightly higher number reporting two or more sexual partners in the preceding year. When analysing the subsample of countries peaking at over 10% prevalence, we reconfirm the main result of an increase in HIV testing at the 10% level of significance (table 5 and online supplemental appendix table S4). Applying a probit model (for binary outcomes) reconfirms our primary results. Online supplemental appendix figures S1, S2 present country-specific IV estimates for each of the 23 countries in the sample, alongside 95% CIs. The majority of point estimates are consistent in direction with the pooled estimate reported in table 3. There is substantial heterogeneity in both the magnitude and precision of estimates across countries. Countries with narrow CIs—indicating stronger identification and larger effective sample sizes—tend to cluster around modest effects, broadly consistent with the pooled result. A small number of countries exhibit extremely wide CIs, reflecting small sample sizes and/or weak first-stage identification in these settings rather than genuine uncertainty about large effects. Finally, our results are robust to correcting for multiple hypothesis testing (table 5).

**Table 5:** Effect of additional year of schooling on primary and behavioural outcomes—primary analysis and robustness checks/subsample analysis

Model number	(1)	(2)	(3)	(4)	(5)	(6)	(7)
Outcome	HIV positive	Ever been tested for HIV	Comprehensive HIV knowledge	Age at first sex	Used condom at last intercourse	2 or more sexual partners in 12 months	Number of sexual partners in lifetime
Primary analysis–pooled sample (coefficient)	−0.005	0.046	0.016	0.198	0.017	0.009	0.078
95% CI	(−0.018 to 0.008)	(−0.001 to 0.094)	(−0.020 to 0.051)	(−0.074 to 0.470)	(−0.005 to 0.039)	(−0.002 to 0.036)	(−0.427 to 0.594)
P value	(0.464)	(0.056)	(0.385)	(0.154)	(0.138)	(0.218)	(0.548)
Adjusted p value	0.381	0.042	0.489	0.1836	0.099	0.327	0.705
Model R1: post 2010 surveys	−0.006	0.056	0.027	0.216	0.007	0.013	0.277
95% CI	(−0.018 to 0.006)	(0.019 to 0.092)	(0.001 to 0.053)	(−0.031 to 0.463)	(−0.015 to 0.029)	(0.001 to 0.026)	(−0.226 to 0.779)
P value	(0.351)	(0.003)	(0.045)	(0.086)	(0.546)	(0.039)	(0.280)
Model R2: 2 window gap	0.003	0.011	0.011	0.134	0.018	0.025	0.158
95% CI	(−0.010 to 0.017)	(−0.030 to 0.052)	(−0.024 to 0.045)	(−0.090 to 0.357)	(−0.004 to 0.040)	(0.001 to 0.048)	(−0.290 to 0.606)
P value	(0.624)	(0.602)	(0.546)	(0.240)	(0.113)	(0.037)	(0.490)
Model R3: high-prevalence countries (>10%)	−0.017	0.074	−0.001	0.579	0.000	0.006	0.280
95% CI	(−0.084 to 0.049)	(−0.002 to 0.150)	(−0.073 to 0.071)	(−0.155 to 1.314)	(−0.043 to 0.042)	(−0.041 to 0.054)	(−0.497 to 1.056)
P value	(0.611)	(0.057)	(0.976)	(0.122)	(0.984)	(0.793)	(0.480)
Model R4: probit model (binary outcomes only)	−0.044	0.157	0.041	—	0.083	0.073	—
95% CI	(−0.165 to 0.077)	(0.073 to 0.240)	(−0.045 to 0.128)	—	(−0.038 to 0.204)	(−0.049 to 0.195)	—
P value	(0.477)	(0.000)	(0.346)	—	(0.178)	(0.240)	—

## Discussion

HIV/AIDS continues to be one of the leading global health threats and sources of premature mortality. Despite progress in reducing HIV incidence and mortality through pharmaceutical interventions, there remains an important need to reduce new infections or increase testing and treatment through social policy means. Exposure to a universal policy reform has often been used as an instrument to undertake causal analysis of schooling on health and development outcomes.^20 22–24 34^ But only three previous studies of this kind were found considering the causal impact of schooling on HIV with national scope. Two of these focused on one country (Botswana) and showed widely contrasting results (either a very large impact on HIV infection or no significant effect for most of the population), while the other focused on two countries (Uganda and Malawi), only including women. Recognising that a much larger number of countries will have had policies expanding education along with national HIV survey data, we focused on 23 countries in Africa, capturing a wide diversity of epidemiological contexts—with national peak adult prevalence rates ranging from under 1% to over 20%. Our analysis includes exposure to both primary and secondary education reforms, including men and women in both rural and urban areas.

In our pooled analysis, we did not find additional years of schooling to have significant impacts on HIV infection across a range of contexts and settings. This was the case even when looking at a subsample of high-prevalence settings. We did find an impact on testing at a 10% significance level, and we found limited evidence of impacts on some sexual behaviours depending on urbanicity or sex. These appear to be driven by the highest prevalence settings. There was no identified impact on knowledge of HIV-related information in the primary analysis. The lack of impact on HIV test positivity indicates that schooling on its own may prove limited as a prevention strategy in many areas. Rather, education may play a broader influential role as a social determinant of health—affecting social position, networks and other structural factors, rather than simply changing individual risk behaviours through knowledge imparted. These social-structural determinants are not easy to evaluate through national surveys or single sector interventions, but provide a rich area for further research to unpack the mechanisms through which education affects HIV risk.

There was no significant impact on comprehensive HIV knowledge, except at a lower 10% threshold for rural areas (see online supplemental appendix table S3). The lack of significance of comprehensive knowledge in the pooled sample may suggest limits to the theory that schooling reduces risk through individual knowledge-driven sexual behaviour change. If schooling is indeed associated with later onset of sexual activity for women, this may support the hypothesis that schools offer ‘safe spaces’ from transmission, although delayed onset could be linked to other factors. Our findings may also support more of a social/structural determinants view of HIV risk, whereby an individual’s education influences the broader environments in which they live, or their future social networks—where norms of testing, condom use and sexual initiation may differ. Data in DHS surveys are limited to explore these concepts in great detail as they lack questions or variables that would enable analysis of many of these effects—resulting in our focus on individual behavioural variables.

A number of factors need to be considered in interpreting these results. First, while we identified policies that expanded education through compulsory schooling, grade expansion or fee removal, it is possible that implementation could have been partial or phased. We attempted to exclude such cases with documentary evidence, but this might not always have been fully identified (table 1 provides references used to judge reforms in each country). Gradual implementation would mean some intervention cohorts may have been only partly exposed, reducing effect sizes. We also pooled both primary and secondary school reforms, which could mask if particular years of school have greater impact for future HIV risk—for example, years when sexual behaviours are developing.

As an IV analysis, there is an assumption that education reforms affect HIV outcomes only through their effect on schooling. While direct effects of such reforms on HIV seem unlikely, there is a chance that such reforms were part of broader policy reforms or social and economic changes that could affect outcomes independently. This is ultimately untestable but it is a common assumption in literature using education reforms as an instrument to study the effects of schooling on health outcomes.^20–24^ We do control for country fixed effects, survey year fixed effects and country-specific cohort trends. Furthermore, reform and survey timings differ across countries, meaning no global change would affect country cohorts in the same way. However, ultimately we cannot completely rule out the possibility that education reforms coincided with other country-level changes that could affect HIV outcomes independently of schooling.

Another concern would be if rates of compliance with HIV testing differ between cohorts, which might reflect testing refusal changing over time. Our online supplemental appendix table S1 shows test refusal rates in 17 countries with multiple survey rounds. In 10 of these countries, testing refusal rates fell in each subsequent round. It is possible that cohort-specific effects could also affect results. Given countries had education reforms and surveys at different times, our control and intervention cohorts would not be attending school at the same points in time in each location. Thus, any universal time-specific changes would likely affect some intervention and some control cohorts depending on country—although our robustness checks included restricting the analysis to surveys conducted post 2010, to capture mature epidemics and available treatment.

While our primary model uses a linear probability model for some binary outcomes, this is consistent with prior causal analyses of this question.^25 35^ We further applied a probit model as a robustness check for binary outcomes, achieving similar results. Ultimately our results find that schooling can have some impacts on HIV-related behaviours such as testing; and potentially sexual debut, recent partners or condom use in specific groups, but impacts may be contextually mediated. We do not find evidence that schooling alone drives reductions in HIV infection rates, however. These results can help to clarify expectations of HIV prevention policy and planning. We also would note that our results do not detract from the established importance of schooling as a global development goal in and of itself, and also as a key determinant of other health outcomes. The difficulty in reproducing early study results for HIV further emphasises the importance of replication efforts and transparency in data and models used in the global health community. Ultimately, our findings contribute to the policy-relevant literature and knowledge on how health and social development are linked, providing a fuller account of the potential impacts of schooling on various elements of HIV risk.

## Supplementary material

10.1136/bmjopen-2025-111925 online supplemental file 1

## Data Availability

Data may be obtained from a third party and are not publicly available.
