# Supplementary material for: Effects of an increase in schooling on HIV infection and behavioural risk: a multicountry instrumental variable analysis of 23 sub-Saharan African countries
Source: BMJ Open. 2026 Aug 4;16(8):e111925. doi: 10.1136/bmjopen-2025-111925 (PMC13448628; doi:10.1136/bmjopen-2025-111925)
Supplement: Supplementary file 1 [file bmjopen-2025-111925.reviewer_comments.pdf]

PEER REVIEW HISTORY

BMJ Open publishes all reviews undertaken for accepted manuscripts. Reviewers are asked to complete a checklist review form and are provided with free text boxes to elaborate on their assessment. These free text comments are reproduced below.

ARTICLE DETAILS

Title (Provisional)

The effects of an increase in schooling on HIV infection and behavioural risk: A multi-country instrumental variable analysis of 23 sub-Saharan African countries

Authors

Parkhurst, Justin; Klaasen, Olivia; Barlow, Pepita; Thomas, Ranjeeta

---

VERSION 1 - REVIEW

**Reviewer**	**1**
**Name**	**Tylleskär, Thorkild**
**Affiliation**	**Universitetet i Bergen, Centre for International health**
**Date**	**29-Nov-2025**
**COI**	**None**

Thank you for allowing me to review this manuscript:

bmjopen-2025-111925

The effects of education on HIV -a multi-country quasi-experimental causal analysis

This manuscript is trying to assess whether education on a macro-level can reduce HIV transmission with a interesting approach. The manuscript is worth publishing after some revisions.

The manuscript is statistically very technical which to some extent goes over my head as a clinically trained person. But I still have some questions and some comments that may improve the manuscript or its accessibility for people like me.

Question:

1) This is an analysis of many settings over several decades. One option would be to analyse each country separately first before making a pooled analysis, using the first half of the equation:

= 0 + 1 + . ##(2 + 3 2

It is not clear to me why this is not done. This could potentially be presented as a forest plot as in Figure 2 and it could visualise any heterogeneity between the data sets.

Other comments:

2) Readers very often start by looking at the figures and tables. Therefore, it is a good idea to have sufficient information in the legend/heading to understand the content of the figure or table. This is nicely done for Figure 1 but not done for Figure 2, several abbreviations are unexplained, and you are even asked to go to the supplement for details.

3) I think the figure in the supplementary material and table S1 are central and should be moved to the main manuscript.

4) In Table 1, one of the column headings is 'Mean/(SD)'. Yet, in this column, not only means (SD) are presented, also proportions as percentages. I do not allow my students to have such mis-matches between column headings and column content in their work.

5) In table 1 it is written 'HIV blood test result' but in the text it is said that 7% were HIV-positive. Should it not read: 'Positive HIV test'?

**Reviewer**	**2**
**Name**	**Rabilloud, Muriel**
**Affiliation**	**Hospices Civils de Lyon, Biostatistique**
**Date**	**14-Dec-2025**
**COI**	**None**

This article aims to quantify the causal effect of increased schooling duration on HIV seropositivity and other outcomes. The authors used an instrumental variable corresponding to the implementation of educational reforms that aimed to increase years of schooling. The statistical analysis strategy is well described and suitable for causal inference. However, the use of OLS models to model binary outcomes is not really suitable, even though this choice was made in order to be able to use models for instrumental variable with 2-stages. At a minimum, a sensitivity analysis should be performed by estimating the marginal effect of years of schooling using a probit or logit model to model the binary outcomes, as done by De Neve et al. Furthermore, this study has seven outcome criteria, and the analysis should account for multiple comparisons by using a method that adjusts the p-values according to the number of comparisons.

VERSION 1 - AUTHOR RESPONSE

Reviewer: 1

1. This is an analysis of many settings over several decades. One option would be to analyse each country separately first before making a pooled analysis, using the first half of the equation:
   $Hi = \beta 0 + \beta 1 Si + i.malei\#\#(\beta 2 agei + \beta 3 age2i$
   It is not clear to me why this is not done. This could potentially be presented as a forest plot as in Figure 2 and it could visualise any heterogeneity between the data sets.

Reply: *Thank you for raising this. Please see our reply to comment #8, above which includes development of the suggested forest plots.*

2. Readers very often start by looking at the figures and tables. Therefore, it is a good idea to have sufficient information in the legend/heading to understand the content of the figure or table. This is nicely done for Figure 1 but not done for Figure 2, several abbreviations are unexplained, and you are even asked to go to the supplement for details.

Reply: *Thank you. We have edited the heading of the table to avoid acronyms. We have a note explaining that data populating the graphs is in the Appendix, but we felt it was not possible to put all those data in the main manuscript so have retained this link to the appendix for additional supplementary information.*

3. I think the figure in the supplementary material and table S1 are central and should be moved to the main manuscript.

Reply: *Thank you for this suggestion, this has been done.*

4. In Table 1, one of the column headings is 'Mean/(SD)'. Yet, in this column, not only means (SD) are presented, also proportions as percentages. I do not allow my students to have such mis-matches between column headings and column content in their work.

Reply: *We appreciate the note on the mismatch. We have edited the headings.*

5. In table 1 it is written 'HIV blood test result' but in the text it is said that 7% were HIV-positive. Should it not read: 'Positive HIV test'?

Reply: *Thank you for spotting this. We have revised the text to say HIV-positive test result.*

Reviewer: 2

6. This article aims to quantify the causal effect of increased schooling duration on HIV seropositivity and other outcomes. The authors used an instrumental variable corresponding to the implementation of educational reforms that aimed to increase years of schooling. The statistical analysis strategy is well described and suitable for causal inference. However, the use of OLS models to model binary outcomes is not really suitable, even though this choice was made in order to be able to use models

for instrumental variable with 2-stages. At a minimum, a sensitivity analysis should be performed by estimating the marginal effect of years of schooling using a probit or logit model to model the binary outcomes, as done by De Neve et al.

Reply*: Thank you for this. We have now included a probit model for binary outcomes as a robustness check with results integrated into the tables and main text – please see reply above to Associate Editor comment #7.*

7. Furthermore, this study has seven outcome criteria, and the analysis should account for multiple comparisons by using a method that adjusts the p-values according to the number of comparisons.

   Reply*: Thank you for suggesting this. We have two families of hypotheses: (1) HIV positive status and testing; (2) Our five behaviour/mechanism outcomes. Consequently, we now include as robustness Romano-Wolf step-down adjusted -values accounting for multiple hypotheses testing within these two sets of families, added as a row in Table 5.*

Clarke, D., Romano, J., & Wolf, M. (2020). The Romano-Wolf multiple hypothesis correction in Stata. *The Stata Journal*, *20*(4). https://doi.org/10.1177/1536867X20976314

VERSION 2 - REVIEW

**Reviewer**	**3**
**Name**	**Dack, Kyle**
**Affiliation**	**University of Bristol, MRC Integrative Epidemiology Unit**
**Date**	**06-Jun-2026**
**COI**	**None**

Statistical review:

This analysis uses an instrumental variable approach (similar to that used in Mendelian Randomization studies) to estimate causal effect from cross-sectional data. This is a valid approach and it is implemented well. My suggestions below are mostly minor to improve clarity and presentation.

Abstract:

1. If space, it would be informative to add information about the time the analysis is relevant to - either the range of DHS survey dates, or the range of birth years.

2. Conclusions - typo "wire"

Methods:

3. It could be helpful (and recommended in STROBE) to state the study design and key elements such as the data source and study dates at the beginning, to provide a high-level summary of the study before going into details.

4. Table 2. In my pdf this is presented within the methods, but it is not referenced until the results. I don't know how this impacts the final formatting but authors may wish to check.

5. Table 2. Typo - static instead of statistics. Are the observations weighted or unweighted? Suggest adding as a footnote or clarifying in the table title.

6. Table 2. The percentages are reported with standard deviation. This is not very interpretable and does not add information because an SD for a binary variable is entirely be determined by the proportion itself. Typically the N and % would be enough to report and might make the table easier to follow.

7. Information about the causal framework, the instrumental variable, assumptions, and rationale is currently spread across the methods. A lot of the explanation is reported at the very end of the methods, along with Figure 1. For readers without knowledge of instrumental variable analyses, it could be difficult to follow the earlier sections of the methods without first understanding the assumed causal framework. Readers may also be unsure or concerned why causality is being inferred from cross-sectional data.

I suggest this could be addressed by moving some IV information to earlier in the paper (e.g. the causal framework, rationale for the IV), plus explanation of how it enables causal inference (e.g. eliminating risk of confounding bias).

8. DHS surveys often present weighted statistics, how was this handled in this analysis?

Results

9. For readers unfamiliar with the method, the association between schooling and educational reform could be elaborated on - it indicates the validity of the instrument along with the later reported F-statistics.

10. Effect estimates: for some outcomes, the unit of the estimate (percentage points change?) should also be reported. It is not clear otherwise what a change of 0.001 HIV positivity means.

11. Table 3: Similar to the above, the effect estimates column contains coefficients with different units, so reporting these would improve the table clarity.

12. Table 4:

-Suggest labelling the table "effect of one year of schooling" or similar to clarify what is being estimated.

-Covariate names: these are Stata variables which are not reader friendly, and could be replaced by plain descriptions, e.g. Age, age squared, country fixed effects, etc.

-However, the covariates are identical for all seven models. It could be more efficient as a footnote.

-The first and fourth rows are not labelled. These could be added, e.g. model number, SE.

-Please clarify estimate units which I think vary per model.

Discussion:

13. The interpretation is reasonable and critically evaluates the findings well. One issue which could be discussed is the plausibility of the exclusion restriction assumption (reform only impacting the outcome via schooling). Direct effects of reform on the outcomes seem unlikely, but a potential risk is if the reform was bundled with other reforms, or otherwise coincided with societal/economic changes which impact the outcomes.

VERSION 2 - AUTHOR RESPONSE

Reviewer 3 Comments:

1. If space, it would be informative to add information about the time the analysis is relevant to - either the range of DHS survey dates, or the range of birth years.

These have been added.

2. Conclusions - typo "wire"

This has been corrected.

Methods:
3. It could be helpful (and recommended in STROBE) to state the study design and key elements such as the data source and study dates at the beginning, to provide a high-level summary of the study before going into details.

This has been added at the start of the section.

4. Table 2. In my pdf this is presented within the methods, but it is not referenced until the results. I don't know how this impacts the final formatting but authors may wish to check.

The table has been moved to the results to align with where it is discussed.

5. Table 2. Typo - static instead of statistics. Are the observations weighted or unweighted? Suggest adding as a footnote or clarifying in the table title.

Corrected and clarified unweighted.

6. Table 2. The percentages are reported with standard deviation. This is not very interpretable and does not add information because an SD for a binary variable is entirely be determined by the proportion itself. Typically the N and % would be enough to report and might make the table easier to follow.

This has been revised in line with the suggestion.

7. Information about the causal framework, the instrumental variable, assumptions, and rationale is currently spread across the methods. A lot of the explanation is reported at the very end of the methods, along with Figure 1. For readers without knowledge of instrumental variable analyses, it could be difficult to follow the earlier sections of the methods without first understanding the assumed causal framework. Readers may also be unsure or concerned why causality is being inferred from cross-sectional data.
I suggest this could be addressed by moving some IV information to earlier in the paper (e.g. the causal framework, rationale for the IV), plus explanation of how it enables causal inference (e.g. eliminating risk of confounding bias).

This has been edited in line with the suggestion, including a short paragraph explaining the logic of the analysis in terms of causality.

8. DHS surveys often present weighted statistics, how was this handled in this analysis?

Noted that unweighted statistics were used given it was not searching for a population-representative estimate.

Results
9. For readers unfamiliar with the method, the association between schooling and educational reform could be elaborated on - it indicates the validity of the instrument along with the later reported F-statistics.

We have added a line explaining that exposure significantly predicted years of schooling.

10. Effect estimates: for some outcomes, the unit of the estimate (percentage points change?) should also be reported. It is not clear otherwise what a change of 0.001 HIV

positivity means.

Text has been edited throughout to clarify percentage point changes where relevant

11. Table 3: Similar to the above, the effect estimates column contains coefficients with different units, so reporting these would improve the table clarity.

The table has been edited for clarity in line with the suggestion

12. Table 4:
-Suggest labelling the table "effect of one year of schooling" or similar to clarify what is being estimated.
-Covariate names: these are Stata variables which are not reader friendly, and could be replaced by plain descriptions, e.g. Age, age squared, country fixed effects, etc.
-However, the covariates are identical for all seven models. It could be more efficient as a footnote.
-The first and fourth rows are not labelled. These could be added, e.g. model number, SE.
-Please clarify estimate units which I think vary per model.

The table has been edited in line with the suggestions

Discussion:
13. The interpretation is reasonable and critically evaluates the findings well. One issue which could be discussed is the plausibility of the exclusion restriction assumption (reform only impacting the outcome via schooling). Direct effects of reform on the outcomes seem unlikely, but a potential risk is if the reform was bundled with other reforms, or otherwise coincided with societal/economic changes which impact the outcomes.

Text has been added to the discussion to address this comment.
