# Supplementary material for: Effects of an increase in schooling on HIV infection and behavioural risk: a multicountry instrumental variable analysis of 23 sub-Saharan African countries
Source: BMJ Open. 2026 Aug 4;16(8):e111925. doi: 10.1136/bmjopen-2025-111925 (PMC13448628; doi:10.1136/bmjopen-2025-111925)
Supplement: Supplementary file 2 [file bmjopen-16-8-s001.docx]

# Supplementary Appendix

In this appendix we provide further details of our searches, statistical methods, and additional relevant information.

## Identification of countries for inclusion

Our country identification strategy started by trying to identify any Low- and Middle-Income Countries (LMICs) which had undertaken significant educational reforms at a national level which could serve as an instrument for the analysis. LMICs were specified as these represent the regions where nationally representative surveys (such as the Demographic Health Surveys) are typically undertaken which can have comparable HIV testing and behavioural survey data available.

The search was strategic – searching for key terms (e.g. ‘compulsory schooling’, ‘universal primary’, etc.) and using reference lists and sources from papers to look for additional sources or country information, and cross referencing with international agency reports or web pages – in particular from the World Bank and UNICEF. Five papers provided information on a large number of countries due to the nature of their analysis,^1-5^ although some only provided lists of countries or lacked details on implementation. At times we had to reply on multiple sources to identify the nature of reform and if implemented at a specific point in time or progressively (we only included those where we could identify clear time break before and after). After this process, we cross referenced our findings against the World Education Reform Database of Stanford University <https://werd.stanford.edu/> which lists education reforms since the 1970s, although with limited contextual informatoin.^6^ Where there were unclear elements – such as the actual start date of a reform, or phased implementation, we would do individual country searches for additional literature of any kind that might provide the most accurate description of reform.

While the survey datasets used are publicly available, they require registration and additional permission requests for HIV data. Before 2025, data could be obtained from the MeasureDHS programme: <https://dhsprogram.com/>; or for Botswana, the data used came from Statistics Botswana: <https://microdata.statsbots.org.bw>. At the time of writing, the DHS website indicates that the DHS programme was ‘on pause’ and no additional datasets could be requested, although existing ones could still be downloaded if previously approved. DHS datasets have numerous methodological reports reflecting on survey strategy, including how potential sources of bias can be considered in individual surveys.^7, 8^ However, given their large sample size and national sampling strategy, they are often considered highly representative of populations.

Table 1 in the main text presents the list of identified countries from this search, indicating: year of reform; type of reform; exit age of children for the final year of schooling affected by the reform; and the birth year cohorts utilised in our analysis. We include the key references used to judge the details of the reforms.

## Testing refusal over time

Another potential source of bias could arise if refusal rates to test for HIV changed over time. We investigated refusal rates for testing over time, to see if there might be large and consistent changes which, in theory, can reflect an unobserved confounder influencing results. In most countries included with multiple rounds of surveys, refusal rates for HIV testing have fallen over time, although not consistently and with some notable exceptions (e.g. Mozambique or Sierra Leone where the latest years had highest refusal rates). The table below provides the country DHS survey years and reported refusal rates for countries with more than one HIV survey round.

**Table S1: HIV test refusal rates by country and DHS survey year (15-49 year olds interviewed)**

|  | DHS Year | Test refusal rate |
| --- | --- | --- |
| Burundi | 2010 | 5.8% |
|  | 2014 | 1.8% |
| Cameroon | 2004 | 5.5% |
|  | 2011 | 4.4% |
|  | 2018 | 2.0% |
| Ethiopia | 2005 | 15.2% |
|  | 2011 | 5.1% |
|  | 2016 | 6.0% |
| Ghana | 2003 | 8.1% |
|  | 2014 | 4.3% |
| Kenya | 2003 | 13.7% |
|  | 2008-9 | 10.8% |
| Lesotho | 2004 | 14.1% |
|  | 2009 | 4.7% |
|  | 2014 | 1.7% |
| Malawi | 2004 | 22.2% |
|  | 2010 | 7.7% |
|  | 2015-16 | 5.1% |
| Mali | 2001 | 12%* |
|  | 2006 | 4.4% |
|  | 2012-13 | 7.6% |
| Mozambique | 2009 | 6.1% |
|  | 2015 | 6.7% |
| Niger | 2006 | 3.5% |
|  | 2012 | 4.2% |
| Rwanda | 2005 | 1.5% |
|  | 2010 | 0.3% |
|  | 2014-15 | 0.5% |
|  | 2019-20 | 0.2% |
| Senegal | 2005 | 12.7% |
|  | 2010-11 | 10.0% |
|  | 2017 | 5.7% |
| Sierra Leone | 2008 | 6.9% |
|  | 2013 | 5.8% |
|  | 2019 | 12.4% |
| Tanzania | 2003-4 | 12.3% |
|  | 2007-8 | 7.1% |
|  | 2011-12 | 7.0% |
| Uganda | 2004-5 | 5.2% |
|  | 2011 | 1.3%** |
| Zambia | 2001-2 | 15.1% |
|  | 2007 | 20.1% |
|  | 2013-14 | 6.8% |
|  | 2018 | 3.9% |
| Zimbabwe | 2005-6 | 15.2% |
|  | 2010-11 | 14.1% |
|  | 2015 | 7.7% |

*Mali 2001 data given to nearest whole number.

**Uganda 2011 data does not include refusal rate for non-interviewed individuals

## Supplementary results

In our main paper we present a selection of findings due to space constraints. Here we present fuller results from our analysis. Tables S2 and S3 show heterogeneity by sex (Male or Female) or residence (Urban or Rural). Table S4 shows main analysis repeated on only those countries with high peak HIV prevalence (over 10%). We further provide forest plots of country-specific instrumental variable analysis estimates for our key outcomes of HIV positive test result and having ever been tested for HIV (Figures S1 and S2).

**Table S2: Heterogeneity by sex in local average treatment effects - second stage estimates**

|  | | | (1) | (2) | (3) | (4) | (5) | (6) | (7) | |
| --- | --- | --- | --- | --- | --- | --- | --- | --- | --- | --- |
|  | | | HIV positive | Comprehensive HIV knowledge | Age at first sex | Used condom at last intercourse | 2 or more sexual partners in 12 months | Number of sexual partners in lifetime | Ever been tested for HIV | |
| Women | | | -0.013 | 0.021 | 0.316** | -0.002 | 0.008 | 0.167 | 0.049* | |
|  | | | (0.009) | (0.020) | (0.143) | (0.012) | (0.005) | (0.188) | (0.027) | |
| Adjusted p-value | | | 0.168 | 0.604 | 0.069 | 0.999 | 0.386 | 0.772 | 0.0693 | |
| Men | | | 0.010 | 0.008 | 0.037 | 0.055** | 0.012 | -0.068 | 0.043 | |
|  | | | (0.011) | (0.029) | (0.225) | (0.026) | (0.019) | (0.654) | (0.033) | |
| Adjusted p-value | | 0.287 | | 0.999 | 0.999 | 0.089 | 0.931 | 0.999 | 0.168 | |
|  | c.Age | | ✓ | ✓ | ✓ | ✓ | ✓ | ✓ | ✓ |  |
|  | c.Age#c.Age | | ✓ | ✓ | ✓ | ✓ | ✓ | ✓ | ✓ |  |
|  | i.Male | | ✓ | ✓ | ✓ | ✓ | ✓ | ✓ | ✓ |  |
|  | c.Birthyear | | ✓ | ✓ | ✓ | ✓ | ✓ | ✓ | ✓ |  |
|  | i.Country | | ✓ | ✓ | ✓ | ✓ | ✓ | ✓ | ✓ |  |
|  | i.Surveyyear | | ✓ | ✓ | ✓ | ✓ | ✓ | ✓ | ✓ |  |
|  | i.Male##(c.Age##c.Age c.Birthyear c.Birthyear#i.Country i. Surveyyear i.Country) | | ✓ | ✓ | ✓ | ✓ | ✓ | ✓ | ✓ |  |
| N | | | 190,116 | 146,925 | 161,745 | 146,508 | 192,730 | 147,039 | 193,287 | |
| F-statistic (Women) | | | 30.104 | 11.939 | 31.511 | 11.367 | 26.480 | 31.854 | 30.657 | |
| F-statistic (Men) | | | 27.852 | 8.411 | 24.288 | 4.114 | 20.027 | 24.429 | 23.681 | |

Notes: Models estimated with two-stage least squares. Coefficients represent percentage point changes. Robust standard errors clustered at the country cohort level in parentheses. Adjusted p-values represent Romano-Wolf p-values adjusted across two families of hypotheses – (1) hiv positive status and testing and. (2) Five behavioural outcomes ^*^ *p* < 0.10, ^**^ *p* < 0.05, ^***^ *p* < 0.01

**Table S3: Heterogeneity by urban-rural in local average treatment effects - second stage estimates**

|  | (1) | (2) | (3) | (4) | (5) | (6) | (7) |
| --- | --- | --- | --- | --- | --- | --- | --- |
|  | HIV positive | Comprehensive HIV knowledge | Age at first sex | Used condom at last intercourse | 2 or more sexual partners in 12 months | Number of sexual partners in lifetime | Ever been tested for HIV |
| Rural | 0.001 | 0.017 | 0.298 | 0.022* | 0.002 | -0.375 | 0.032 |
|  | (0.007) | (0.019) | (0.183) | (0.013) | (0.009) | (0.291) | (0.031) |
| Adjusted p-value | 0.940 | 0.832 | 0.217 | 0.201 | 0.990 | 0.525 | 0.455 |
| Urban | -0.019 | 0.028 | 0.038 | 0.003 | 0.024** | 0.423 | 0.057** |
|  | (0.013) | (0.028) | (0.172) | (0.021) | (0.012) | (0.494) | (0.023) |
| Adjusted p-value | 0.227 | 0.782 | 0.990 | 0.990 | 0.079 | 0.832 | 0.013 |
| c.Age | ✓ | ✓ | ✓ | ✓ | ✓ | ✓ | ✓ |
| c.Age#c.Age | ✓ | ✓ | ✓ | ✓ | ✓ | ✓ | ✓ |
| i.Male | ✓ | ✓ | ✓ | ✓ | ✓ | ✓ | ✓ |
| i.Urban | ✓ | ✓ | ✓ | ✓ | ✓ | ✓ | ✓ |
| c.Birthyear | ✓ | ✓ | ✓ | ✓ | ✓ | ✓ | ✓ |
| i.Country | ✓ | ✓ | ✓ | ✓ | ✓ | ✓ | ✓ |
| i.Surveyyear | ✓ | ✓ | ✓ | ✓ | ✓ | ✓ | ✓ |
| i.urban##(c.Age##c.Age i.male c.Birthyear c.Birthyear#i.Country i. Surveyyear i.Country) | ✓ | ✓ | ✓ | ✓ | ✓ | ✓ | ✓ |
| N | 187,342 | 142,977 | 158,218 | 143,495 | 189,722 | 147,039 | 189,321 |
| F statistic (Rural) | 35.843 | 27.471 | 23.822 | 23.697 | 34.053 | 26.090 | 17.548 |
| F statistic (Urban) | 9.509 | 15.740 | 6.413 | 11.099 | 19.446 | 13.111 | 9.637 |

Notes: Models estimated with two-stage least squares. Coefficients represent percentage point changes. Robust standard errors clustered at the country cohort level in parentheses. Adjusted p-values represent Romano-Wolf p-values adjusted across two families of hypotheses – (1) hiv positive status and testing and. (2) Five behavioural outcomes ^*^ *p* < 0.10, ^**^ *p* < 0.05, ^***^ *p* < 0.01

**Table S4: Local average treatment effects in sub-sample of countries with prevalence above 10% - second stage estimates**

|  | (1) | (2) | (3) | (4) | (5) | (6) | (7) |
| --- | --- | --- | --- | --- | --- | --- | --- |
|  | HIV positive | Comprehensive HIV knowledge | Age at first sex | Used condom at last intercourse | 2 or more sexual partners in 12 months | Number of sexual partners in lifetime | Ever been tested for HIV |
| High prevalence (>=10%) | -0.017 | -0.001 | 0.579 | -0.000 | 0.006 | 0.280 | 0.074* |
|  | (0.611) | (0.976) | (0.122) | (0.984) | (0.793) | (0.480) | (0.057) |
| c.Age | ✓ | ✓ | ✓ | ✓ | ✓ | ✓ | ✓ |
| c.Age#c.Age | ✓ | ✓ | ✓ | ✓ | ✓ | ✓ | ✓ |
| i.Male | ✓ | ✓ | ✓ | ✓ | ✓ | ✓ | ✓ |
| c.Birthyear | ✓ | ✓ | ✓ | ✓ | ✓ | ✓ | ✓ |
| i.Country | ✓ | ✓ | ✓ | ✓ | ✓ | ✓ | ✓ |
| i.Surveyyear | ✓ | ✓ | ✓ | ✓ | ✓ | ✓ | ✓ |
| i.Male##(c.Age##c.Age c.Birthyear c.Birthyear#i.Country i. Surveyyear i.Country) | ✓ | ✓ | ✓ | ✓ | ✓ | ✓ | ✓ |
| N | 70,336 | 59,579 | 63,905 | 58,664 | 72,198 | 57,141 | 73,388 |
| F statistic | 6.886 | 6.216 | 6.142 | 8.620 | 6.304 | 7.967 | 6.948 |

Notes: Models estimated with two-stage least squares. Coefficients represent percentage point changes. Robust standard errors clustered at the country cohort level in parentheses. ^*^ *p* < 0.10, ^**^ *p* < 0.05, ^***^ *p* < 0.01

**Figure S1: Forest plot of country-specific instrumental variable results for the impact of schooling on positive HIV test result.**

**Figure S2: Forest plot of country-specific instrumental variable results for the impact of schooling on HIV testing.**

## Higher prevalence settings

To see if results may differ in countries with higher background prevalence or more severe HIV epidemics, we identified the peak HIV prevalence in adults (age 15-49) from aidsinfo.unaids.org, which provides comparable data from 1990 onwards. We classified countries as low (under 5%), medium (5 to 10%) or higher (over 10%) HIV prevalence. Table S5 below lists peak prevalence rates per included country. In the main results we present findings from comparing countries with peak prevalence above 10% to those below.

**Table S5: Peak national adult HIV prevalence for included countries**

|  | Peak Prevalence (%) age 15-49 | Low, Med, or High? |
| --- | --- | --- |
| Angola | 1.9 | L |
| Botswana | 26.1 | H |
| Burundi | 5.1 | M |
| Cameroon | 6.9 * | M |
| Ethiopia | 3.2 | L |
| Gambia | 2.0 | L |
| Ghana | 2.6 | L |
| Kenya | 10.2 | H |
| Lesotho | 24.7 | H |
| Liberia | 3.0 | L |
| Malawi | 15.4 | H |
| Mali | 1.8 | L |
| Mozambique | 12.6 | H |
| Niger | 0.8 | L |
| Republic of the Congo | 5.2 | M |
| Rwanda | 5.2 | M |
| Senegal | 0.7 | L |
| Sierra Leone | 1.6 | L |
| South Africa | 18.7 | H |
| Tanzania | 6.8 | M |
| Uganda | 10.9 | H |
| Zambia | 14.3 | H |
| Zimbabwe | 25.6 | H |

*Estimate from UNAIDS 2003 Fact Sheet. No data available at aidsinfo.unaids.org.

## Appendix References

1.	Carr OG. Promoting priorities: Explaining the adoption of compulsory schooling laws in Africa. International Journal of Educational Development. 2022;88:102523.

2.	Diaz-Serrano L. The duration of compulsory education and the transition to secondary education: Panel data evidence from low-income countries. International Journal of Educational Development. 2020;75:102189.

3.	Edmonds EV, Shrestha M. The impact of minimum age of employment regulation on child labor and schooling. IZA Journal of Labor Policy. 2012;1:1-28.

4.	Koski A, Strumpf EC, Kaufman JS, Frank J, Heymann J, Nandi A. The impact of eliminating primary school tuition fees on child marriage in sub-Saharan Africa: A quasi-experimental evaluation of policy changes in 8 countries. PloS one. 2018;13(5):e0197928.

5.	Tennant B. A comparative analysis of developing countries and their progress toward universal primary education: University of South Dakota; 2007.

6.	Bromley P, Kijima R, Overbey L, Furuta J, Choi M, Santos H, et al. World Education Reform Database (WERD). V3 ed: Harvard Dataverse; 2023. 10.7910/DVN/C0TWXM.

7.	Pullum TW. Strategies to assess the quality of DHS data. Rockville, Maryland, USA: ICF; 2019.

8.	Elksabi M, Khan A. Modeling interviewer effects in DHS surveys. Rockville, Maryland, USA: ICF; 2022.

9.	Stanford Graduate School of Education. World Education Reform Database 2024 [Available from: <https://werd.stanford.edu/>.

10.	De Neve J-W, Fink G, Subramanian SV, Moyo S, Bor J. Length of secondary schooling and risk of HIV infection in Botswana: evidence from a natural experiment. The Lancet Global Health. 2015;3(8):e470-e7. 10.1016/S2214-109X(15)00087-X.

11.	Sommeiller E, Wodon Q. Enrolment gains from the elimination of primary school user fees in Burundi. Washington, DC: World Bank. 2014.

12.	Njie H, Manion C, Badjie M. Girls' Familial Responsibilities and Schooling in The Gambia. International Education Studies. 2015;8(10):48-62.

13.	Nishimura M, Ogawa K, Sifuna D, Chimombo J, Kunje D, Ampiah J, et al. A Comparative Analysis of Universal Primary Education Policy In Ghana, Kenya, Malawi and Uganda. 2009.

14.	Ekundayo OS. The right to free and compulsory primary education in Ghana: lessons for other African countries. Journal of Law, Policy, and Globalization. 2018;69:105.

15.	Chicoine L. Education and fertility: Evidence from a policy change in Kenya. 2012.

16.	Sifuna DN. The challenge of increasing access and improving quality: An analysis of universal primary education interventions in Kenya and Tanzania since the 1970s. International Review of Education. 2007;53:687-99.

17.	Somerset A. Access to Primary Education in Kenya: A case study of the impact of the 2003 FPE initiative at nine schools in Nairobi City and Nyeri District. University of Sussex, Centre for International Education 2008.

18.	Wakano A, Yamada H. Cost-sharing policy and its long-term impact on quantity–quality tradeoff: Evidence from Kenya. 2024.

19.	Morojele P. Rural teachers’ views: What are gender-based challenges facing Free Primary Education in Lesotho? South African Journal of Education. 2013;33(3).

20.	Andriano L, Monden CW. The causal effect of maternal education on child mortality: Evidence from a quasi-experiment in Malawi and Uganda. Demography. 2019;56(5):1765-90. 10.1007/s13524-019-00812-3.

21.	Bleck J, Guindo BM. Education for all, education for whom, education for what? Lessons from Mali. Development in Practice. 2013;23(8):1007-21. 10.1080/09614524.2013.839983.

22.	author N. Malawi: progress towards education for all. Available through UNESCO digital library; Unclear date.

23.	ActionAid. Low quality of public schools drives parents towards private education in Mozambique. Johannesburg: ActionAid International Secretariat; 2017.

24.	Republic of the Congo. Constitution of the Republic of the Congo. 1992.

25.	Nkurunziza J, Broekhuis A, Hooimeijer P. Free Education in Rwanda: Just One Step towards Reducing Gender and Sibling Inequalities. Education Research International. 2012;2012(1):396019. <https://doi.org/10.1155/2012/396019>.

26.	Grogan L. Universal Primary Education and School Entry in Uganda. Journal of African Economies. 2008;18(2):183-211. 10.1093/jae/ejn015.

27.	Right to Education Project. Right to education country factsheet: Sierra Leone. 2016.

28.	World Bank. Education in Sierra Leone: Present challenges, future opportunities. Washington D.C.: The World Bank; 2007. Report No.: 0821368680.

29.	Asim S, Chugunov D, Gera R. Fiscal Implications of Free Education: The Case of Tanzania: World Bank; 2019.

30.	Deininger K. Does cost of schooling affect enrollment by the poor? Universal primary education in Uganda. Economics of Education review. 2003;22(3):291-305.

31.	Riddell A. The Introduction of free primary education in sub-Saharan Africa. Background paper prepared for the Education for All Global Monitoring Report 2003/4 Gender and Education for All: The Leap to Equality. 2003. Report No.: 2004/ED/EFA/MRT/PI/62

32.	Ukoh J. The Cost of Zambia's Educational System: Afrcain Leadership Magazine; 2023 [Available from: <https://www.africanleadershipmagazine.co.uk/the-cost-of-zambias-educational-system/>.

33.	Grépin KA, Bharadwaj P. Maternal education and child mortality in Zimbabwe. J Health Econ. 2015;44:97-117. 10.1016/j.jhealeco.2015.08.003.

34.	Machingaidze T, Pfukani P, Shumba S. The quality of primary education: some policy suggestions based on a survey of schools. Paris: Institute for Educational Planning, Ministry of Education and Culture, Zimbabwe, and UNESCO; 1998.

35.	Nhundu TJ. A Decade of Educational Expansion in Zimbabwe: Causes, Consequences, and Policy Contradictions. The Journal of Negro Education. 1992;61(1):78-98. 10.2307/2295630.
